# Follicular Fluid Proteomic Analysis to Identify Predictive Markers of Normal Embryonic Development

**DOI:** 10.3390/ijms25158431

**Published:** 2024-08-01

**Authors:** Janusz Przewocki, Dominik Kossiński, Adam Łukaszuk, Grzegorz Jakiel, Izabela Wocławek-Potocka, Stanisław Ołdziej, Krzysztof Łukaszuk

**Affiliations:** 1 Institute of Mathematics, University of Gdansk, 80-308 Gdańsk, Poland; 2 iYoni App—For Fertility Treatment, LifeBite, 10-763 Olsztyn, Poland; d.kossinski@iyoni.app (D.K.); luka@gumed.edu.pl (K.Ł.); 3 Edinburgh Medical School, College of Medicine and Veterinary Medicine, The University of Edinburgh, 47 Little France Crescent, Edinburgh EH25 9RG, UK; 4 Invicta Research and Development Center, 81-740 Sopot, Poland; 5 First Department of Obstetrics and Gynaecology, Centre of Postgraduate Medical Education, 01-004 Warsaw, Poland; 6 Department of Gamete and Embryo Biology, Institute of Animal Reproduction and Food Research, Polish Academy of Sciences, 10-748 Olsztyn, Poland; i.woclawek-potocka@pan.olsztyn.pl; 7 Intercollegiate Faculty of Biotechnology UG & MUG, University of Gdańsk, Abrahama 58, 80-307 Gdańsk, Poland; stanislaw.oldziej@biotech.ug.edu.pl; 8 Department of Obstetrics and Gynecology Nursing, Medical University of Gdańsk, 80-210 Gdańsk, Poland

**Keywords:** follicular fluid, proteomics, mass spectrometry, embryo quality, oocyte quality, immunoglobulin heavy constant alpha 1, keratin type II cytoskeletal 1 protein, dickkopf-related protein 3, heat shock cognate 71 kDa protein

## Abstract

Ageing populations, mass “baby-free” policies and children born to mothers at the age at which they are biologically expected to become grandmothers are growing problems in most developed societies. Therefore, any opportunity to improve the quality of infertility treatments seems important for the survival of societies. The possibility of indirectly studying the quality of developing oocytes by examining their follicular fluids (hFFs) offers new opportunities for progress in our understanding the processes of final oocyte maturation and, consequently, for predicting the quality of the resulting embryos and personalising their culture. Using mass spectrometry, we studied follicular fluids collected individually during in vitro fertilisation and compared their composition with the quality of the resulting embryos. We analysed 110 follicular fluids from 50 oocyte donors, from which we obtained 44 high-quality, 39 medium-quality, and 27 low-quality embryos. We identified 2182 proteins by Sequential Window Acquisition of all Theoretical Mass Spectra (SWATH-MS) using a TripleTOF 5600+ hybrid mass spectrometer, of which 484 were suitable for quantification. We were able to identify several proteins whose concentrations varied between the follicular fluids of different oocytes from the same patient and between patients. Among them, the most important appear to be immunoglobulin heavy constant alpha 1 (IgA1hc) and dickkopf-related protein 3. The first one is found at higher concentrations in hFFs from which oocytes develop into poor-quality embryos, the other one exhibits the opposite pattern. None of these have, so far, had any specific links to fertility disorders. In light of these findings, these proteins should be considered a primary target for research aimed at developing a diagnostic tool for oocyte quality control and pre-fertilisation screening. This is particularly important in cases where the fertilisation of each egg is not an option for ethical or other reasons, or in countries where it is prohibited by law.

## 1. Introduction

A reliable assessment of the developmental potential of oocytes before fertilisation could significantly change the picture of reproductive medicine. According to the World Health Organisation, around 17.5% of couples suffer from infertility, with more than 12% at the point of fertilisation. It is believed that more than 186 million women currently have fertility problems worldwide. Similarly, the decision to have a first child has been significantly delayed across the world, exceeding 31 years of age in developed countries at the time of childbirth.

Aside from the increasingly widespread fashion for “baby-free” policies among young adults in developed countries and rare cases of congenital infertility, most fertility problems are age-related. The causes of the age- and lifestyle-related infertility epidemic include genetic defects in embryos, decreased sperm parameters, decreased ovarian reserves, endometriosis, polycystic ovarian syndrome, the hostility of cervical mucus, implantation disorders, the obstruction of the fallopian tubes, and submucosal myomas. Age-related infertility is associated with diseases that are virtually non-existent in youth and therefore do not interfere with achieving pregnancy at the physiologically intended time. This is supported by data from Hutterian reproduction, where early efforts to get pregnant, the lack of premarital sex, and the lack of influence of an economic factor on the decision to obtain a pregnancy result in a fertility problem affecting only about 2% of couples [1].

Due to delayed attempts at pregnancy and the associated factors causing couples to have lower fertility, in vitro fertilisation is becoming an increasingly common treatment option. It is the most effective and sometimes the only possible method of treatment. However, its effectiveness is still far from our expectations, with only limited progress over the past 20 years. According to its key performance indicators (KPIs) [2] only 75–90% of the oocytes retrieved during the in vitro fertilisation procedure are at the correct stage of development. Only about 80% of them will undergo fertilisation, of which 70% will develop to the cleavage stage by day 3 and 60% will develop to a blastocyst, of which only about 60% will be of so-called top quality (TQ). As a result, we still lack the tools to initially assess the quality of collected oocytes. The availability of such information would significantly enhance the clinical decision-making process, allowing the right number of cells to be fertilised and helping to predict their development. Furthermore, it would also help to identify the exact problems affecting individual oocytes, paving the way for their personalised culture and ultimately improving the quality of the embryos produced.

Several non-invasive methods for assessing oocytes and embryos are currently being studied. Among these, the most commonly used, mainly due to its low cost and widespread availability, is the assessment of embryo morphology performed by embryologists or using automated methods. Metabolomics is also being used experimentally. The metabolic profiling of culture media containing human oocytes can provide information on the metabolic state of the cells, although this requires the integration of automated, high-throughput, real-time metabolomic assessments with microfluidic platforms. However, the most promising is the analysis of the human follicular fluid (hFF) proteome, which can provide a set of indicators of oocyte health based on the presence or absence of specific proteins. It is considered the most promising because of the identification and quantification of hundreds of proteins in a single assay, providing a broad picture of the biological state of the oocytes [3]. Proteins are key effectors of cellular function. Unlike genomics, their presence and concentrations directly affect the functionality of the oocyte and its ability to develop into further stages. Previous studies have shown that specific protein profiles in hFF can be correlated with oocyte quality and pregnancy outcomes, offering direct and functional links with oocyte developmental competence [4,5]. Unfortunately, most studies to date have failed to address two limitations of follicular fluid spectrometric studies—their cost and the availability of fluid samples identified and linked to the embryonic development of the originating oocyte. As a result, most of these studies have been based on small patient groups and samples of their follicular fluid obtained from the largest follicle or from pooled follicular fluids from a given patient.

It therefore seems crucial to obtain information linking the proteomic composition of the follicular fluid with the quality of the oocyte and its development after fertilisation. Hence, the aim of this study was to separately examine obtained follicular fluids and identified oocytes and to assess the subsequent development of embryos derived from them.

## 2. Results

### 2.1. Clinical Data of Donors and Quality of the Obtained Embryos

The population of patients included in the study were healthy oocyte donors aged between 18 and 35 years old. The clinical characteristics of the population are presented in Table 1. Each donor underwent evaluation in accordance with FDA-mandated regulations, which included extensive screening for genetic disease carriers and the exclusion of gene rearrangement diseases through peripheral blood leukocyte karyotyping.

### 2.2. Classifications of All Proteins Identified in the Follicular Fluids

Of the 2182 proteins that were identified in the studied hFFs, 484 were quantified. They were classified based on the Panther database [6] into the following protein classes: defence/immunity protein—18.9% (92 genes), metabolite interconversion enzyme—12.9% (62 genes), and protein-modifying enzyme—10.2% (49 genes). The proportion of unclassified proteins was 10.2% (49 proteins). A complete list of the identified classes can be found in Table A1. The molecular functions of the identified proteins were mostly binding—160 genes—and catalytic activity—112 genes—as seen in Table A2.

On the basis of biological processes, the quantified proteins were classified as follows: cellular processes—33.1% (159 genes), response to stimulus—28.7% (138 genes), metabolic processes—27% (130 genes), and biological regulation—25.8% (124 genes). Other biological processes whose proteins have been identified can be found in Table A3. A total of 29.9% (144 genes) could not be classified.

The spectrum of the metabolic pathways was the widest—Table A4. As many as 77.1% (371) of the proteins were not identified in any metabolic process, with the remaining most involved in blood coagulation, 5.8% (28 proteins); the integrin signalling pathway, 3.3% (16 proteins); the gonadotropin-releasing hormone receptor signalling pathway, 2.1% (10 proteins); the plasminogen activating cascade, 1.5% (7 proteins); and the Wnt signalling pathway, 1.5% (7 proteins).

### 2.3. Proteins Associated with Embryo Quality

#### 2.3.1. Classification of Embryo Quality

The hFF samples obtained were classified according to embryo quality, which was assessed on the basis of specific developmental features identified by microscopic imaging. For standardisation purposes, the assessment was performed at Day 5 and Day 6 using the grading system described in the Istanbul criteria [7]. In the context of this study, embryos that had reached the blastocyst stage by day 5 and received a grade 1 trophectoderm were described as being of good quality. Moreover, embryos reaching the blastocyst stage by day 6, as well as those exhibiting a grade 2 trophectoderm at day 5 or 6, were classified as being of fair quality. All embryos that did not fulfil any of the above requirements were assigned to the poor-quality subgroup. As a result, out of a total of 110 embryos sampled, 44 were classified as good quality, 39 as fair quality, and 27 as poor quality.

#### 2.3.2. Machine Learning-Based Proteomic Analysis

Random Forest classifiers were employed to attempt to distinguish the three subgroups of embryonic quality based on their measured protein abundances. The classifiers utilised a Gini impurity as the impurity measure (cf. Section 4.5), defined for any set of samples *Q* in the following way:

(1) $$H\left(Q\right)=\sum_{i=1}^{3}p_{i}\left(1-p_{i}\right),$$

where $p_{i}$ denotes the relative frequency of samples with class *i* in *Q* (here the index *i* indicates one of “good”, “fair”, or “poor”).

The Random Forest consisted of 50 trees with a maximum depth of 3 to prevent overfitting. Additionally, the stopping criterion was that the minimum number of samples per leaf was 30 and the sample weights were inversely proportional to their respective class sizes. The RFECV algorithm was executed for 30 cycles to calculate the protein scores. Balanced accuracy was used to score the resulting models.

**Figure 1:**
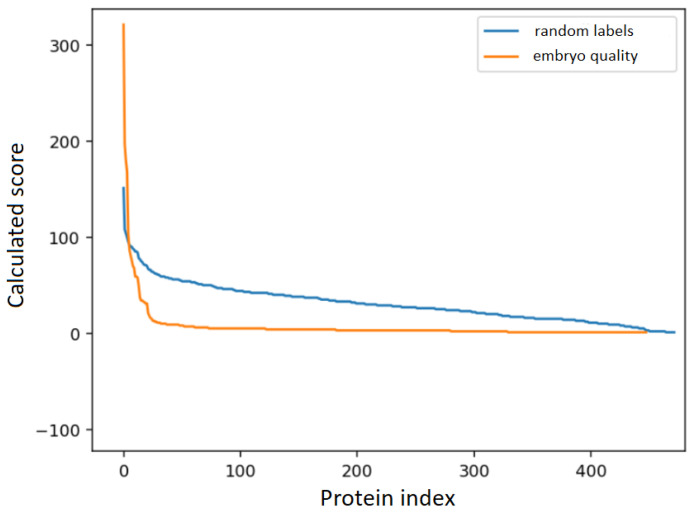
Protein scores calculated with the RFECV algorithm. A comparison of the results obtained with our target variable (embryo quality) and the random assignment of labels.

The derived protein scores were sorted and are shown in Figure 1. Notably, approximately 20 proteins exhibited disproportionately high scores, standing out from the linear trend observed for irrelevant proteins. This finding therefore indicates that the concentration of these 20 proteins varies between follicular fluids associated with blastocysts of different qualities, determined during a morphological assessment (Table 2). Compared with the follicular fluids of oocytes giving rise to embryos with a poor morphology ($FF_{poor}$), follicular fluids from oocytes associated with blastocysts with a good morphology ($FF_{good}$) contained, among other proteins, higher levels of dickkopf-related protein 3 and heat shock cognate 71 kDa protein. In contrast, $FF_{poor}$ were found to have more keratin type II cytoskeletal 1, immunoglobulin heavy constant alpha 1, pyruvate kinase PKM, transforming growth factor-beta-induced protein, multimerin-2, and platelet glycoprotein Ib alpha chain. Oocytes whose follicular fluids gave rise to blastocysts of an intermediate morphology ($FF_{fair}$) were associated with the lowest values of peptidyl-prolyl cis-trans isomerase B, alpha-mannosidase 2, transforming growth factor-beta-induced protein, ectonucleotide pyrophosphatase/phosphodiesterase 2, immunoglobulin heavy constant alpha 1, and moesin. In addition, they also exhibited the highest values of heterogeneous nuclear ribonucleoproteins C1/C2. Amongst the 20 proteins identified, dickkopf-related protein 3 appeared to be the most significant marker of morphological alterations, as it was associated with the greatest degree of change in abundance between $FF_{good}$ and $FF_{poor}$ and the highest protein score value.

**Figure A1:**
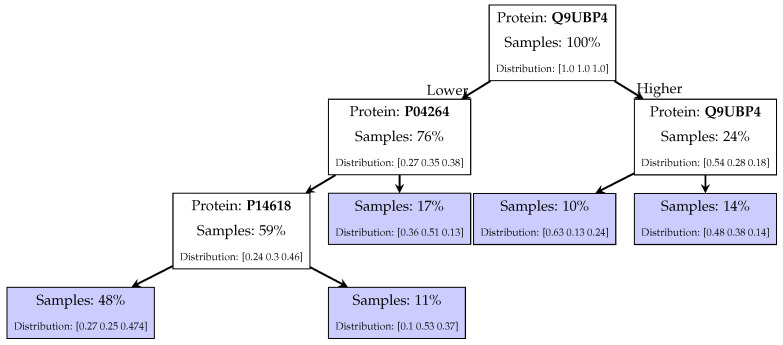
Selected classification tree with dickkopf-related protein 3 as the main node; the conditional distributions in the description of the nodes refer to the probabilities of “good”, “fair”, and “poor” samples, respectively.

The following steps in the analysis focused on identifying significant differences in the abundance of each hFF protein between individual patients. This was achieved by first applying logarithms to the protein abundance values and then calculating their median from each set of biological replicates taken from a given follicular fluid sample. The median values obtained were further grouped according to patient ID numbers and compared using a one-way ANOVA analysis. As determined by this statistical test, the abundance of dickkopf-related protein 3 was significantly associated with certain patients (adjusted-$R^{2}$ = 0.3, F-test *p*-value = 0.009, no significant heteroscedasticity detected after the analysis of residuals; residuals followed normal distribution). This was further complemented by an analysis of a single decision tree, in which dickkopf-related protein 3 was utilised as the top predictor (see Figure A1). As such, the model acted to classify the hFF samples into those with a high or a low abundance of dickkopf-related protein 3 according to a specific threshold value that was learned from the data during the training process. Most interestingly, the hFF samples with high amounts of dickkopf-related protein 3 were found to be more likely to give rise to well—rather than poorly— morphologically developed embryos. The comparison between $FF_{good}$ and $FF_{poor}$ yielded an OR = 2.8 and the result was borderline significant with a *p*-value = 0.07, as determined from the interaction term in the Poisson model explaining the observed sample counts in terms of embryo quality and the indicator of high or low protein abundance.

**Figure A2:**
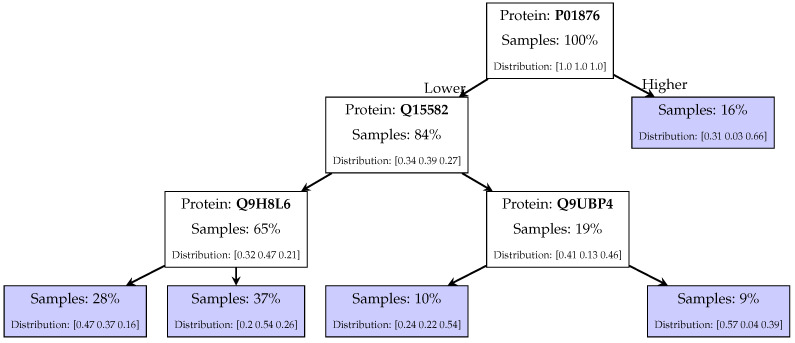
Selected classification tree with immunoglobulin heavy chain alpha as the main node; the conditional distributions in the description of the notes refer to the probabilities of “good”, “fair” and “poor” samples, respectively.

Furthermore, an analogous analysis of the abundance of immunoglobulin heavy constant alpha 1 in the hFF samples revealed some additional significant findings. The amount of the evaluated protein type was found to exhibit a high degree of association with specific patients (adjusted-$R^{2}$ = 0.95, F-test *p*-value < 0.0001, no significant heteroscedasticity detected after analysis of residuals; residuals followed normal distribution). This relationship was considerably stronger than that found in case of dickkopf-related protein 3, which is likely to reflect the sevenfold higher abundance of immunoglobulin heavy constant alpha 1 compared to dickkopf-related protein 3 in hFF samples and the consequently smaller degree of error associated with those measurements. When immunoglobulin heavy constant alpha 1 was used as the top predictor in a decision tree model (see Figure A2), samples of hFF classified as having a high abundance of the protein were more likely to lead to the adverse—rather than good—morphological development of the embryo. The comparison between $FF_{good}$ and $FF_{poor}$ yielded an OR = 0.32 with a *p*-value < 0.001, as determined from the interaction term in the Poisson model explaining the observed sample counts in terms of embryo quality and the indicator of high or low protein abundance.

The effectiveness of a prediction model for the three classes “good”, “fair”, and “poor” based on the identified set of 20 proteins was estimated to be approximately 42% (we applied a cross-validation scheme identical to the one used in the RFECV algorithm).

It is crucial to emphasise that even a completely random large set of protein abundances can show patterns purely by chance. In traditional statistical methods, this problem is mitigated by controlling for the false discovery rate. Here, to make sure that our results are not random, we compared the calculated scores with the results obtained from the zero distribution (i.e., the distribution where all features are irrelevant). These scores were derived from the same features, but with their class labels randomly permuted. The comparison shown in Figure 1 indicates that the set of proteins is far from random, as evidenced by the different shape of the score curve. This observation is in line with the results of the cross-validation, where the calculated balanced accuracy is different from random (i.e., the expected 33%).

We noticed that only four proteins have scores higher than those calculated from the zero distribution. However, it is important to note that score values are not independent. Due to the design of the algorithm, statistically significant proteins will have high score values at the expense of the scores of irrelevant proteins, resulting in the irrelevant proteins having much lower scores compared to those in the zero distribution.

### 2.4. Protein–Protein Interactions

**Figure 2:**
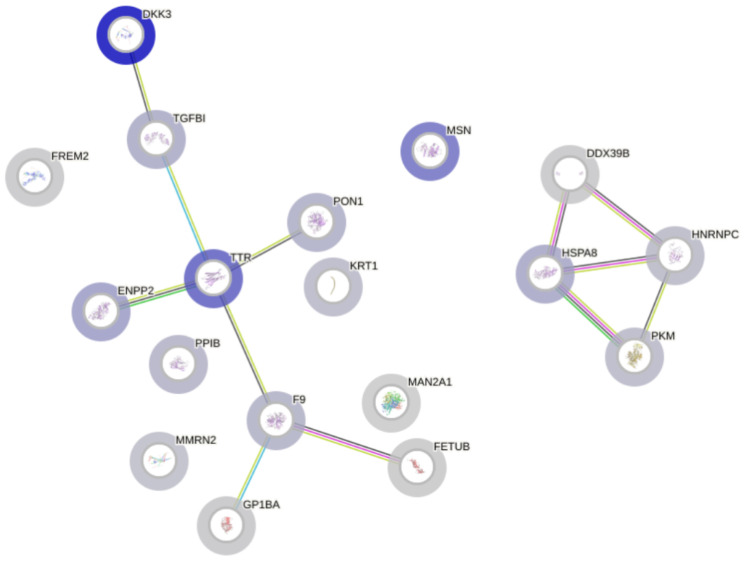
A protein–protein interaction network functional enrichment analysis created using STRING v.12.0. Predicted interactions were summarised using coloured lines: gene co-expression by a black line, gene neighbourhood by a green line, experimental evidence by a purple line, database evidence by a light blue line, and text-mining evidence by a yellow line.

In order to expand the scope of our analysis, the identified list of 20 hFF proteins was inserted into the STRING Network Software v. 12.0 to identify significant interactions between proteins within this subset [8]. Such relationships can allow proteins to perform tasks that individual proteins cannot perform on their own, making protein–protein interactions an essential component of many biochemical cascades and cellular functions. As presented in Figure 2, a key protein identified in the network was transthyretin (TT) which assists in the transport of thyroxine and retinol within the developing embryo [9]. Maintaining adequate levels of the former molecule is known to be essential for proper neurogenesis (the differentiation and maturation of neurons, myelination, and the formation of synaptic connections), the regulation of cellular growth processes, and bone development [10]. Retinol, on the other hand, influences the spatial and temporal patterns of the expression of specific genes, which are particularly relevant for the formation of the foetal heart and eyes [11]. In addition, a relationship between TT and ectonucleotide pyrophosphatase/phosphodiesterase (ENPP2) was highlighted by the network. The function of ENPP2 focuses on the regulation of lipid metabolism pathways and may therefore influence the composition of cellular membranes, as well as signalling pathways, during embryonic development [12].

Furthermore, a significant degree of interaction was identified between heat shock protein family A member 8 (HSPA8) and pyruvate kinase M2 (PKM), which are key components of cellular stress responses and energy metabolism, respectively. HSPA8 has previously been shown to play a vital role in preventing inappropriate protein folding during the synthesis and preservation of protein structures under conditions of cellular stress—both of these protective effects seem to be of significant importance during embryonic development, which is characterised by a high rate of cell division and rapid metabolic changes [13].

## 3. Discussion

In the present study, we investigated the relationship between the composition of the proteins detected and quantified in the follicular fluids and the development of embryos from the derived oocytes. We studied healthy oocyte donors whose cells were fertilised. The male factor is very important and genetically affects 50% of the material. It is less important for the metabolism of the embryo, especially up until the third day of development. After that, full genome activation begins and the influence of the sperm’s DNA becomes visible.

For this reason, we treated the male factor in our study very restrictively. We excluded any patient whose semen parameters deviated from the norms described in the WHO Manual 2021. We also took into consideration the issue of sperm DNA fragmentation, which, in our experience, has a major impact on embryo development. We implemented the TUNEL method based on cytometry. Whilst the generally accepted norm is less than 15%, in this paper we adopted a value of 12% based on our own observations, as we have observed that up to this level of fragmentation we obtain the best embryos.

In principle, three scenarios can be considered: studying the whole material; removing the most abundant proteins so as not to obscure the signal of the less abundant proteins; and focusing on the most regulatory proteins, such as growth factors, hormones, and key regulators of metabolic pathways, using labelled proteins, for which we are preparing an analysis. For our study, we opted for the middle ground—removing proteins with a significant quantitative advantage by immunodepleting approximately 94% of a total of 14 proteins (albumin, IgG, antitrypsin, IgA, transferrin, haptoglobin, fibrinogen, alpha2-macroglobulin, alpha1-acid glycoprotein, IgM, apolipoprotein AI, apolipoprotein AII, complement C3, and transthyretin). We decided to conduct research in this direction because we wanted to obtain a broad overview of the proteins detectable in hFF with the intention of evaluating them in the context of predicting the quality of the oocytes and the resulting embryos. This result has been achieved, as we identified more than 2000 proteins using this approach, and the creation of such a large collection is a very good result compared to the literature data on hFF [4,14,15,16]. In order to avoid problems arising from even small shifts in the chromatogram, calibration peptides (iRT peptides) were added to each sample. However, by removing a significant amount of the above proteins, it was possible to quantify and compare the remaining proteins in the study groups.

This also made it possible to study subtle differences in protein abundance in hFFs which are associated with embryonic development. The decision to use the Random Forest to analyse this type of data was based on its ability to detect weak signals in noisy data and also on several methodological considerations related to the nature of mass spectrometry data.

Firstly, this type of data frequently contains outliers, which can significantly disrupt the performance of many classification algorithms. Random Forests inherently mitigate the impact of outliers due to their ensemble approach, where the aggregation of multiple decision trees reduces the influence of any single aberrant data point on the final prediction [17]. Moreover, the distribution of protein abundances in our dataset varies considerably, with many proteins exhibiting log-normal distributions, while others do not conform to any specific parametric form. Random Forests, as a nonparametric method, do not impose assumptions about the data’s distribution, making them particularly suitable for this heterogeneity. Additionally, Random Forests provide interpretable models through various feature importance metrics, enabling a clearer understanding of the influence of different proteins on the classification outcomes.

In the field of bioinformatics, it is common to encounter datasets characterised by a large number of features and a relatively small number of samples, often including irrelevant variables. Random Forests excel in such high-dimensional settings by effectively managing and utilising a large number of input variables. This adaptability is crucial for enhancing predictive accuracy in scenarios with many irrelevant or noisy features.

Random Forests also demonstrate robust predictive performance in the presence of predominantly noisy variables. The ensemble nature of this algorithm helps in reducing the risk of overfitting, as the errors of individual trees tend to cancel each other out, thereby increasing generalisability of the model. Their consistently high predictive power has positioned Random Forests among the top-performing algorithms in various comparative evaluations. Their ability to extract meaningful insights from complex and noisy biological datasets highlights their utility and effectiveness in bioinformatics research. This performance parity, combined with the added benefits of their interpretability and feature selection, underscores the suitability of Random Forests for tasks requiring both high accuracy and transparency in decision making [18]. The efficacy of Random Forests in classifying biological data is well documented. Numerous studies have successfully applied this method to classify and analyse various types of biological datasets, validating its robustness and reliability in the domain of bioinformatics [18,19,20,21]. Finally, Random Forests facilitate feature selection, which is crucial for identifying the most relevant genes or proteins associated with different biological categories. Methods such as Recursive Feature Elimination (RFE) provide valuable insights into the most influential features within a dataset, aiding in the interpretation and understanding of bioinformatics data [22,23,24].

Our findings highlighted several key relationships between protein abundance in hFFs and embryo quality. Dickkopf-related protein 3 was most abundant in hFFs associated with the highest quality embryos. In contrast, immunoglobulin heavy constant alpha 1 and moesin were most abundant in hFFs associated with poor-quality embryos. Transthyretin had the lowest abundance in hFFs associated with fair-quality embryos.

Interestingly, some proteins, including transthyrethin, exhibited their lowest/highest abundance in hFFs associated with fair-quality embryos, but higher/lower levels with both good- and poor-quality embryos. This surprising observation may be explained by differences in the biological processes that influence the trophectoderm’s quality (which is associated with fair embryo quality) versus those impacting overall blastocyst development.

Significant differences in protein abundance were observed between the hFFs from different patients. In some cases, follicular fluids from the same patient had very similar levels of certain proteins, such as immunoglobulin heavy constant alpha 1. In other cases, significant variance was not related to individual patients, as seen with dickkopf-related protein 3. This pattern might be due to differences in protein abundance and associated relative measurement error differences, which warrants further investigation.

Unfortunately, most studies to date have failed address two limitations of the spectrometric testing of follicular fluid—its cost and the availability of the fluid identified and linked to the embryonic development of the originating oocyte. Studies to date have relied on the examination of a single follicular fluid from the largest follicle or of pooled follicular fluid from a given patient. This introduces two types of bias—when testing fluid from the largest follicle, only one fluid is tested, and this may often not be representative. Ovulation stimulation is often an art of compromise between the number of oocytes obtained and their quality. Quite often, it is necessary to sacrifice the largest follicles (which have exceeded their optimum size and thus stage of development) to allow the growth of a greater number of smaller follicles that still need time to mature. Hence, the fluid obtained may come from a follicle with a worse-than-average prognostic status. At the same time, the development of the embryos derived from these fluids is not followed, resulting in the loss of a direct link between the test result and the experimental outcome. On the other hand, combining hFFs does not allow the results to be linked to embryo development (except in rare cases where all follicles develop equally), while introducing a lot of contamination into the study due to mixing fluids containing oocytes at completely different stages of development.

Therefore, the main strength of our study is the material analysed. We were able to collect hFFs from individual ovarian follicles, label them unambiguously, and link them to oocyte quality and development after fertilisation through their individual culture. This allowed us not only to assess the differences in hFF composition between individual donors, but also to investigate the variability in protein composition between individual follicles within the same organism.

A limitation of our study, as with most proteomics studies, is the number of samples tested. Nevertheless, we examined 110 samples, in biological triplicate, from 50 oocyte donors, which is sufficient to start looking for protein differences between the hFFs from oocytes from which we obtained embryos of different qualities. Additionally, the chosen laboratory workflow for the proteomic studies included several factors, such as immunodepletion effects and peptide ion suppression, which could have affected the accuracy of protein quantification. The subsequent analysis used the Random Forest algorithm, which tends to exclude highly correlated features. These sources of bias may have led to the omission of some biomarkers in our study.

The evaluation of follicular fluids requires further research and the results should be collected in databases for comparative re-analysis. Therefore, it seems important to collect individual follicular fluids and to observe the developing embryos derived from them. This will make it possible to modify their stimulation according to its progress and to individualise the culture media according to the metabolic state of the retrieved oocyte.

## 4. Materials and Methods

### 4.1. Flow Chart of Patient Recruitment and Fluid Collection and Examination

The study, designed in 2019, was conducted at the Medical University of Gdansk and the Invicta fertility clinics. Donors were deemed to be eligible for the study when it was known that the cells would be fertilised with semen meeting the WHO standards. The women were qualified for in vitro fertilisation due to their willingness to be egg donors. The exclusion criteria were as follows: patients under 18 years of age and over 35 years of age; a sperm donor with reduced semen parameters (below WHO 5th edition standards [25]); and sperm DNA fragmentation, determined cytometrically by the TUNEL method, above 12%. Cases with abnormal oocyte fertilisation results in previous cycles were also excluded. Due to the pandemic and difficulties in accessing the material, sample collection was extended until early 2023. We recruited 75 egg donors to this study. During follicular fluid collection, in 21 cases individual follicular fluids could not be completely separated from each other. We excluded these cases from further testing. In four additional cases, there were doubts about the compatibility of individual oocytes with their follicular fluids due to possible mislabelling. We finally included 50 donors in the study, and we individually collected a total of 388 cumuli and secured follicular fluids from a minimum of 2 and a maximum of 11 of their follicles. We studied 110 follicular fluids from 50 donors, with 2 to 3 fluids per donor (see Table 3).

The experiments conducted are part of the project entitled “Identification of Biomarkers of Early Embryonic Development and Pregnancy”, which was approved by the Independent Bioethics Committee at the Medical University of Gdansk (decision 62/2016). All oocyte donors were informed about the protocol and consented to participating in the study. Their written consent obtained also included their permission to publish data related to their treatment, provided that patient anonymity was maintained.

### 4.2. IVF Procedure and Embryo Development

#### 4.2.1. Stimulation

All patients were treated with in vitro fertilisation (IVF) using short-protocol stimulation [26]. Before starting stimulation, ultrasound and hormonal tests were performed to exclude the presence of dominant follicles and to verify that peripheral blood hormone levels were as follows: oestradiol below 50 pg/mL, LH below 6 mIU/mL, and progesterone below 0.5 ng/mL Once the effect of a premature recruitment of the dominant follicle had been ruled out, stimulation with gonadotropins was initiated. Menopausal gonadotropins (Menopur, Ferring) with equal FSH and LH activity were used. Dosing was based on the patient’s baseline AMH level (in the range of 150 to 225 IU per day) with 0.05 mg triptorelin administered subcutaneously from the first day of stimulation. On the eighth day of stimulation, the stimulation dose was adjusted to prepare for oocyte retrieval. Stimulation was terminated after obtaining at least 3 follicles with a diameter of more than 18 mm, with the administration of 5000 IU of hCG intramuscularly (Pregnyl, MSD) for final oocyte maturation 36 h before oocyte retrieval.

#### 4.2.2. Oocyte Retrieval (Pick Up) and Collection of Samples

The oocyte retrieval procedure was performed under brief general anaesthesia with Propofol and Fentanyl. Oocytes were retrieved using disposable oocyte retrieval needles (Gynemed, Sierksdorf, Germany) under the control of ultrasound images obtained using the IC-9-RS vaginal transducer and the GE Voluson P6. The fluid collected from the ovarian follicles was immediately transferred to the embryologist, who continuously reported on the cumuli obtained so far (clusters of granulosa cells from the released ovarian thalamus that may contain an oocyte). If no oocyte was obtained from a given follicle, the attempt was repeated by rinsing the follicle with the same fluid and retrieving it again. After the procedure, the samples were filtered through a 5 μm mesh at room temperature to remove the erythrocytes, white blood cells, and granulosa cells. The fluid was collected and stored at −20 °C for further analysis. The oocytes were kept separately and labelled with the same number as the collected and frozen fluid.

#### 4.2.3. Embryo Culture

The cumuli obtained were stored under conditions of 6% ${\mathrm{CO}}_{2}$ and low oxygen pressure (5% $O_{2}$) in 37 °C in incubators (Labotect C18) inside laminar chambers (Lamil 90 or 120). All oocytes were stripped of their surrounding granulosa cells—they were subjected to decoronisation—2 to 5 h after collection. Their maturity was then graded on a scale: mature cells in the metaphase of their second meiotic division (MII), immature cells in metaphase of their first meiotic division (MI), immature cells at the germinal vesicle (GV) stage, overripe—atretic—cells, and no oocyte in the cumulus. Only mature cells were fertilised. Immature cells, on the other hand, were subjected to further culture in oocyte maturation medium. After one day, their maturity was assessed and additional mature cells were fertilised. In vitro fertilisation was performed by micromanipulation (intracytoplasmic sperm injection—ICSI). The systems used consisted of Nikon Te2000S, U, or E inverted microscopes equipped with Hoffman modulation contrast using Eppendorf NK2 micromanipulators. Heating tables (Okolab, Pozzuoli, Italy) were used to provide full heating of the surface of the ICSI dishes mounted on three-plate microscope tables. Micromanipulator pumps from Eppendorf (Leipzig, Germany); an air pump to hold the egg (CellTram Air), and an oil pump with extra precision to deliver the sperm into the oocyte (CellTram vario) were also used. The entire procedure was carried out with full video documentation, which was analysed by the embryology team as part of the quality control activities of the procedure.

After fertilisation, the cells were cultured in Labotect C18 incubators for a further 5 to 7 days until full maturation—blastocyst formation—or developmental arrest and the onset of apoptosis. Their culture was performed in G1 and G2 sequencing media (Vitrolife, Gothenburg, Sweden). Embryos were assessed on day 1 of culture—the evaluation of fertilisation and rejection of abnormally fertilised cells, day 3—the evaluation of cell divisions (Cummins classification [27]), and day 5—blastocyst maturity (Istanbul criteria).

### 4.3. Sample Preparation

The experiments included comparative qualitative and quantitative studies and spectral library preparation for the SWATH-MS quantification on our samples. The process of optimising the sample preparation method and instruments’ operation was carried out in several steps. The entire process is summarised in Table 4. In brief, after thawing, the hFF was additionally centrifuged at 1000× *g* for 10 min to separate all morphological structures (cellular debris). Working on a chromatographic system with microfluidics, we had to take additional steps to obtain as many proteins as possible for the library. We used a MARS 14 column (Agilent, Santa Clara, CA, USA) to immunodeplete proteins present at high concentrations. The samples were not fractionated. Protein concentrations were measured using a spectrophotometer by quantifying their absorbance at 280 nm Protein material was digested with FASP (tripsin) (1:50 enzyme to protein weight ratio) using a standard Filter-Aided Sample Preparation procedure (FASP) [28] on a Microcon with 30 kDa of cut-off membrane (Merck-Millipore, Burlington, MA, USA). The Multienzyme Digestion (MED) FASP procedure involved three consecutive digestions with LysC (1:50), trypsin (1:100), and chymotrypsin (1:100) (all enzymes from Promega Corporation, Madison, WI, USA). First, the hFF was lysed using a buffer containing 1% sodium dodecyl sulphate (SDS) and 50 mMdithiothreitol (DTT) in 100 mM Tris-HCl of pH for 8 for 10 min at 95 °C. (all reagents from Sigma-Aldrich, St. Louis, MO, USA). A total of 100 μg of protein was applied to each filter. Briefly, the filters were washed several times with a buffer containing 8 M urea in 100 mM Tris-HCl pH 8.5 by centrifugation at 10,000×*g* for 20 min Proteins were alkylated with 55 mM iodoacetamide (IAA, Sigma-Aldrich, St. Louis, MO, USA) for 20 min at room temperature in the dark. Finally, traces of IAA and urea were washed away with 100 mM Tris-HCl pH 8.5 and the enzyme was added to the filters for overnight digestion at 37 °C. The resulting peptides were eluted with 100 mM Tris-HCl pH 8.5. In the case of MED-FASP, the filters were placed in new tubes and the digestion and elution steps were repeated with different enzymes. Digestion with chymotrypsin was carried out for 3 h in a buffer containing 10 mM CaCl_2_ in 100 mM Tris-HCl pH 7.8. The resulting proteolytic peptides were fractionated by RP-HPLC (Reversed-Phase High-Performance Liquid Chromatography) at high pHs and desalted using the STAGE (STop And Go Extraction) tip procedure [29] on in-house prepared tips filled with C18 solid phase (3M™ Empore™, St. Paul, MN, USA). Briefly, 10 μg of peptide was added to the tip, which was previously equilibrated with 1% acetic acid in water. After washing, the peptides were eluted with a buffer containing 60% acetonitrile (ACN)/1% acetic acid in water and evaporated in a SpeedVac to obtain volumes ready for Mass Spectrometry (MS) measurements (5 μL for Q Exactive HF-X or 10 μL for Triple TOF 5600+). To avoid problems caused by even small shifts in the chromatogram, calibration peptides (iRT peptides) were added to each sample. The iRT (indexed retention time) kit (Biognosys, Zurich, Switzerland) was spiked with samples used for SWATH-MS spectral library preparation or SWATH-MS quantification at a 1:10 standard to sample volume ratio to perform retention time calibration. This allowed for the generation of a collection of over 2000 proteins.

### 4.4. LC-MS/MS Measurements and Quantitative Data Processing

The LC-MS/MS measurements for the Triple Quad-TOF workflow were acquired on the TripleTOF 5600+ hybrid mass spectrometer with a DuoSpray Ion Source (AB SCIEX, Framingham, MA, USA) coupled with the Eksigent microLC (Ekspert MicroLC 200 Plus System, Eksigent, Redwood City, CA, USA). Samples were loaded onto the LC column using the CTC Pal Autosampler (CTC Analytics AG, Zwinger, Switzerland), using a 5 μL injection. Buffers A and B constituted of 0.1% (*v*/*v*) formic acid in water and ACN, respectively. LC separations were performed on the ChromXP C18CL column (3 μm, 120 Å, 150×0.3 mm; Eksigent, Redwood City, CA, USA) using a gradient of 8–40% Buffer B over 30 min with a flowrate of 5 μL/min. All measurements were performed in a positive ion mode. The system was controlled by the Analyst TF 1.7.1 software (AB SCIEX, Framingham, MA, USA). Data-dependent acquisition (DDA) analyses consisted of a 250 ms TOF survey scan in the *m*/*z* range of 400–1000 Da followed by a 100 ms Product Ion scan in the *m*/*z* range of 100–1500 Da, which resulted in a 2.3 s cycle time. The top 20 candidate ions with charge states from 2 to 5 were selected for collision-induced dissociation (CID) fragmentation with rolling collision energy. Former target ions were excluded after 2 occurrences for 5 s SWATH-MS [30] analyses were performed in a looped product ion mode. A set of 25 variable-width windows was constructed via equalized ion frequency distribution with the use of SWATHTuner [31] to cover the *m*/*z* range of 400–1000 Da The collision energy of each window was calculated for +2 to +5 charged ions centred on the window, with a spread of 5. The SWATH-MS1 survey scan was acquired in high-sensitivity mode in the range of 400–1000 Da at the beginning of each cycle, with an accumulation time of 50 ms, and it was followed by 40 ms accumulation time high-sensitivity product ion scans, which resulted in a total cycle time of 1.1 s The database search for spectral library construction was performed in ProteinPilot 4.5 software (AB SCIEX, Framingham, MA, USA) using the Paragon algorithm against the SwissProt Homo sapiens database (ver. 26.07.2019; 20,428 entries) merged with the iRT standard sequence and the following parameters: a TripleTOF 5600+ instrument (AB SCIEX, Framingham, MA, USA); the alkylation of cysteines by iodoacetamide; trypsin enzyme digestion, an ID focus on biological modifications; the search effort “thorough ID”; and a threshold of detected proteins [Conf] > 10%. The resulting group file was loaded into MS/MS All with SWATH Acquisition MicroApp 2.01 in PeakView 2.2 (AB SCIEX, Framingham, MA, USA) to automatically create a spectral library with the following set parameters: modified peptides allowed and shared peptides excluded. The library was processed via SWATH-MS measurements of the samples. Retention time calibration was performed manually with the use of iRT kit peptides. The maximum number of peptides per protein was 6 and the extracted ion chromatogram (XIC) parameters were set to a 10 min extraction window width and 75 ppm XIC width. The sample preparation workflow and the final results are summarised in Table 4.

There were two normalisation steps involved. First, the spectra of individual samples were normalised in MarkerView using total area sums. Finally, in the second step, SWATH-MS intensities were normalised in Perseus at the level of all samples.

### 4.5. The Random Forest Algorithm

The protein abundances obtained from the SWATH-MS workflow were analysed using the Random Forest classifier, which is a versatile and powerful ensemble learning algorithm. Its primary purpose is to create a classification scheme for samples based on features (such as protein abundances) in order to predict associated labels (e.g., embryo quality; see Section 2.3.1).

The algorithm works by constructing a multitude of decision trees during training, each trained on a different random subset of the dataset. By combining the predictions of these individual trees through averaging, it enhances predictive accuracy and mitigates the risk of overfitting.

A single decision tree within the Random Forest ensemble is constructed using a process that involves recursively partitioning the input feature space based on the values of different features. Here we present an overview of how a single tree is created:

**Initialization:** The tree starts with a root node that contains a random subset of $m_{0}$ training samples:

(2) $$\begin{array}{c} Q_{0}=\{\left(x,y,w\right)|x\mathrm{is}a\mathrm{vector}\mathrm{of}\mathrm{features},\\ \;y\mathrm{is}\mathrm{an}\mathrm{associated}\mathrm{label}\mathrm{and}w\mathrm{is}\mathrm{the}\mathrm{weight}\mathrm{of}\mathrm{the}\mathrm{sample}\}. \end{array}$$

**Best feature selection:** Let us denote the subset of samples under consideration in the training phase of the *m*th node as $Q_{m}$. First, a random subset $F_{m}$ containing *n* features is created (hyperparameter *n* is kept fixed throughout training and is usually set to be the square root of the number of all features). In the next step, the best of the selected features is found based on a chosen criterion (e.g., a Gini impurity or entropy, cf., Section 2.3.1) called impurity function *H*.

**Splitting:** The set of samples $Q_{m}$ is split into two subsets—$Q_{m}^{left}=\left\{\left(x,y,w\right)|x_{i}<t_{m,i}\right\}$ and $Q_{m}^{right}=Q_{m}\setminus Q_{m}^{left}$. The feature $x_{i}\in F_{m}$ and the threshold $t_{m,i}$ are selected to minimise the mean impurity:

(3) $$G\left(Q_{m}\right)=\frac{w_{m}^{left}}{w_{m}}H\left(Q_{m}^{left}\right)+\frac{w_{m}^{right}}{w_{m}}H\left(Q_{m}^{right}\right),$$

where

(4) $$w_{m}=\sum_{\left(x,y,w\right)\in Q_{m}}w,\;w_{m}^{left}=\sum_{\left(x,y,w\right)\in Q_{m}^{left}}w,\;w_{m}^{right}=\sum_{\left(x,y,w\right)\in Q_{m}^{right}}w.$$

The goal of the chosen criterion metric *H* is to maximise the homogeneity of the target variable (i.e., ideally within each subset we would like to have samples belonging mostly to the same class). This process of splitting the dataset $Q_{m}$ yields two new nodes of the decision tree and is repeated recursively for each subset $Q_{m}^{left}$ and $Q_{m}^{right}$ until a stopping criterion is met.

**Stopping Criterion:** The recursion stops when one of the following conditions is met:

1. The maximum tree depth is reached;
2. The number of samples in the current node falls below a certain threshold;
3. Further splitting does not lead to significant improvement in the chosen metric.

**Leaf Nodes:** Once the stopping criterion is met, the current node becomes a leaf node, and it is assigned a probability distribution based on the distribution of labels.

When making predictions for a new sample using a single decision tree within a Random Forest ensemble, one of the two following steps are typically followed.

**Traversal:** The new sample is passed down the tree starting from the root node. At each node, the tree evaluates a specific feature of the sample based on the splitting threshold $t_{m,i}$ learned during training. Then, the sample is directed either to the left or right child node of the current node. This process continues recursively, with the sample traversing down the tree from one node to another until it reaches a leaf node.

**Leaf Node Prediction:** Once the sample reaches a leaf node, the tree assigns a probability distribution associated with the node. If our goal is to predict a single class based on the feature, the class with the highest probability is taken.

It is important to note that each decision tree in the Random Forest ensemble makes an independent prediction for the new sample. In classification tasks, the final prediction of the Random Forest classifier is determined by aggregating the predictions of all the trees in the ensemble by averaging (note that this approach is slightly different from the original one, where majority voting is used, see [32]). An advantage of Random Forests is their ability to rank features by assigning importance to each feature. Typically, the Mean Decrease in Impurity (MDI) is used as an estimate of feature importance. This can be defined separately for each feature *x* in every individual tree *T*:

(5) $$MDI_{T}\left(x\right)=\sum_{m\in T}p\left(m\right)\Delta i\left(m\right).$$

The above sum is calculated over the nodes *m* splitting the samples $Q_{m}$ into the two subsets $Q_{m}^{left}$ and $Q_{m}^{right}$ and using feature *x* in their splitting criterion. Then, the decrease in impurity Δi(m) for node *m* is calculated to be

(6) $$\Delta \left(m\right)=H\left(Q_{m}\right)-\frac{w_{m}^{left}}{w_{m}}H\left(Q_{m}^{left}\right)-\frac{w_{m}^{right}}{w_{m}}H\left(Q_{m}^{right}\right).$$

Next, the weight p(m) of each node considered in the sum is defined as:

(7) $$p\left(m\right)=\frac{w_{m}}{w},$$

where *w* denotes the sum of the weights associated with all samples in the training dataset.

Finally, the feature importance of *x* over the whole Random Forest is defined by simply averaging all $MDI_{T}\left(x\right)$ for all trees *T* in the ensemble.

### 4.6. Recursive Features’ Elimination with Cross-Validation—Algorithm Description

In our analysis, we employed a version of RFECV (Recursive Feature Elimination with Cross-Validation) implemented in the Python library scikit-learn v1.2.1. This method requires the classifier used to be capable of computing feature importances, a criterion met by the Random Forest classifier (cf. Section 4.5). Below is a brief description of the algorithm:

1. **Data Partitioning:** The data are divided into folds, where each fold uses samples from one patient as test data and the remaining samples to train the classifier. The number of folds equals the number of patients, ensuring each patient’s samples are used as test data exactly once.
2. **Feature Elimination:** For each fold, the RFE algorithm begins by iteratively removing features. First, the classifier is fitted to compute feature importances. Then, the least important feature is removed, and the model’s score is calculated using the fold’s test data. This process is repeated until only one feature remains.
3. **Score Averaging:** The scores calculated for each fold and each number of features during step 2 are averaged to obtain mean scores as a function of the number of features. The optimal number of features, $n_{features}$, is defined as the number with the highest mean score.
4. **Final Model Fitting:** Finally, the classifier is fitted over the entire dataset, and the $n_{features}$ with the highest importance are selected.
5. **Iteration:** Steps 1 to 4 are repeated a predefined number of times or until only one feature remains.

This algorithm also allows us to assign scores to the selected features. At each iteration in step 4, a subset of features is selected. When a feature is selected, its score is incremented by one. Thus, features that persist longer throughout the iterations will accumulate higher scores.

An original version of the RFE approach evaluates feature importance using a support vector machine (SVM) model, selecting features for elimination based on their ranked importance [33]. This method can also be adapted for other models such as Random Forests (RFs), which have intrinsic mechanisms for evaluating feature importance [34,35,36].

## 5. Conclusions

In conclusion, our study of the composition of individually retrieved oocytes and their follicular fluids, derived from embryos of different qualities, showed that the composition of their fluids differed depending on the quality of the final developed blastocysts. We identified the differential abundances of 20 proteins, including immunoglobulin heavy constant alpha 1 (IgA1hc) and dickkopf-related protein 3. Although these changes were modest, with the difference of their averages being around 20–30%, our analysis showed that these subtle differences could lead to significant variations at the extremes of their distribution. Specifically, the Random Forest algorithm indicated that extreme abundances of certain proteins could dramatically affect the prognosis of embryo development, doubling the odds of either a good or poor outcome. These findings open up new opportunities for further translational research into the significance of the proteins that differentiate blastocysts of different qualities.

## Figures and Tables

**Table 1:** A summary of the clinical characteristics of the oocyte donors; values are means (standard deviation).

Variable	Results
No. subjects	50
Age (y)	27.4 (2.3)
BMI (kg/m^2^)	21.7 (3.3)
AMH (ng/mL)	2.8 (1.5)
Inhibin B	62.3 (30.8)
Day 3 basal FSH	6.3 (4.3)
Day 3 basal LH	7.2 (5.3)
Day 3 basal oestradiol	43.2 (30.4)
DHEAS	226 (72.5)
Testosterone	1.5 (1.7)
SHBG	74.2 (35.4)
AFC	16.7 (6.2)

**Table 2:** Twenty most significant proteins with fold changes in their median protein abundances (samples from $FF_{poor}$ embryos were taken as the reference).

Protein Name	Uniprot ID	Score	$\log_{2}{\mathrm{FC}}_{\mathrm{good}}$	$\log_{2}{\mathrm{FC}}_{\mathrm{fair}}$
Dickkopf-related protein 3	Q9UBP4	321.0	0.43	−0.00047
Transthyretin	P02766	197.0	−0.075	−0.29
Immunoglobulin heavy constant alpha 1	P01876	180.0	−0.26	−0.34
Moesin	P26038	168.0	−0.15	−0.31
Ectonucleotide pyrophosphatase/phosphodiesterase 2	Q13822	103.0	0.17	−0.35
Heat shock cognate 71 kDa protein	P11142	87.0	0.36	0.23
Transforming growth factor-beta-induced protein ig-h3	Q15582	81.0	−0.19	−0.36
Serum paraoxonase/arylesterase 1	P27169	76.0	−0.1	−0.27
Coagulation factor IX	P00740	70.0	−0.06	−0.28
Peptidyl-prolyl cis-trans isomerase B	P23284	68.0	0.02	−0.53
Keratin, type II cytoskeletal 1	P04264	59.0	−0.36	−0.39
Pyruvate kinase PKM	P14618	59.0	−0.24	0.09
Heterogeneous nuclear ribonucleoproteins C1/C2	P07910	58.0	0.18	0.57
Multimerin-2	Q9H8L6	49.0	−0.27	0.04
Platelet glycoprotein Ib alpha chain	P07359	37.0	−0.24	−0.21
FRAS1-related extracellular matrix protein 2	Q5SZK8	34.0	0.09	−0.058
Spliceosome RNA helicase DDX39B	Q13838	34.0	0.22	0.06
Fetuin-B	Q9UGM5	33.0	0.072	0.09
Immunoglobulin kappa variable 6D-21	A0A0A0MT36	32.0	−0.11	−0.083
Alpha-mannosidase 2	Q16706	31.0	−0.31	−0.49

**Table 3:** Flow chart of patient recruitment.

Recruitment	
75 patients	
Follicular fluids taken separately	Mixed follicular fluids
54 patients	21 patients
Confirmed identification of follicular fluid and associated embryo	Lack of certain identification of follicular fluids and their oocytes
50 patients	4 patients
Collected follicular fluids and cells	
223	
Number of follicular fluids tested (maximum 3 per patient)	
110	
Number of biological repeats	
330 (110×3)	

**Table 4:** Optimisation of sample preparation and SWATH analysis conditions for the TripleTOF spectrometer.

Steps		
Protein fractionation for the library		high-pH RP-HPLC, Immunodepletion
Fractionation of peptides for quantitative analysis		No
Digestion method		FASP (trypsin)
Peptide purification		C18 Stage Tips
Method parameters LC		30 min, 8–40% buffer B
Parameters	Data-dependent acquisition (DDA) MS	400–1000 Da, 250 ms
	MS/MS	100–1500 Da, 100 ms
	Cycle time	2.3 s
SWATH Method parameters	MS	400–1000 Da, 50 ms
	MS/MS	10–1500 Da, 40 ms
	Cycle time	1.1 s
Transmission windows		25 window variables in range 400–1000 Da
Results		
Total number of proteins identified in the experiments		2182
Number of proteins identified in fractions HMWF/LMWF		2177/14
Number of proteins identified after ultrafiltration		129
Number of quantified proteins		484
Number of proteins quantified with CV < 20%		98

## Data Availability

The datasets presented in this article are not readily available because the data are part of an ongoing study. Requests to access the datasets should be directed to janusz.przewocki@ug.edu.pl.

## Abbreviations

The following abbreviations are used in this manuscript:

AFC	antral follicles count
AMH	anti-Mullerian hormone
BMI	body mass index
DDA	Data-dependent acquisition
DHEAS	dehydroepiandrosterone-sulfate
E2	oestradiol
FASP	Filter-Aided Sample Preparation
FSH	follicle-stimulating hormone
HMWF	High-Molecular-Weight Fraction
hFF	human follicular fluid
LC	Liquid Chromatography
LH	luteinizing hormone
LMWF	Low-Molecular-Weight Fraction
MS	Mass Spectrometry
RP-HPLC	Reversed-Phase High-Performance Liquid Chromatography
SHBG	sex hormone-binding globulin

## Appendix A

**Table A1:** Proteins according to their protein classes.

Protein Class	Number of Genes	Proportion of Identified Proteins
defence/immunity protein	91	18.9%
metabolite interconversion enzyme	62	12.9%
protein-modifying enzyme	49	10.2%
unclassified	49	10.2%
protein-binding activity modulator	44	9.1%
cytoskeletal protein	24	5.0%
cell adhesion molecule	23	4.8%
transfer/carrier protein	23	4.8%
extracellular matrix protein	20	4.2%
chaperone	15	3.1%
transmembrane signal receptor	13	2.7%
intercellular signal molecule	12	2.5%
scaffold/adaptor protein	11	2.3%
RNA metabolism protein	10	2.1%
calcium-binding protein	9	1.9%
membrane traffic protein	8	1.7%
translational protein	6	1.2%
transporter	4	0.8%
chromatin/chromatin-binding or -regulatory protein	3	0.6%
structural protein	2	0.4%
DNA metabolism protein	2	0.4%
gene-specific transcriptional regulator	1	0.2%

**Table A2:** Proteins classified according to their molecular function.

Molecular Function	Number of Genes	Proportion of Identified Proteins
unclassified	195	40.5%
binding	160	33.3%
catalytic activity	112	23.3%
molecular function regulator activity	32	6.7%
structural molecule activity	19	4.0%
molecular transducer activity	11	2.3%
antioxidant activity	7	1.5%
transporter activity	6	1.2%
ATP-dependent activity	4	0.8%
translation regulator activity	2	0.4%
cargo receptor activity	1	0.2%
cytoskeletal motor activity	1	0.2%

**Table A3:** Proteins classified according to their biological function.

Biological Process	Number of Genes	Proportion of Identified Proteins
cellular process	159	33.1%
unclassified	144	29.9%
response to stimulus	138	28.7%
metabolic process	130	27.0%
biological regulation	124	25.8%
immune system process	88	18.3%
multicellular organismal process	53	11.0%
developmental process	44	9.1%
localization	22	4.6%
biological process involved in interspecies interaction between organisms	17	3.5%
homeostatic process	7	1.5%
locomotion	5	1.0%
detoxification	2	0.4%
growth	2	0.4%
reproductive process	1	0.2%
reproduction	1	0.2%

**Table A4:** Proteins classified with respect to their function in metabolic pathways.

Metabolic Pathway	Number of Genes	Proportion of Identified Proteins
Unclassified	371	77.1%
Blood coagulation	28	5.8%
Integrin signalling pathway	16	3.3%
Gonadotropin-releasing hormone receptor pathway	10	2.1%
Plasminogen activating cascade	7	1.5%
Wnt signalling pathway	7	1.5%
Glycolysis	6	1.2%
Cadherin signalling pathway	6	1.2%
Alzheimer disease-presenilin pathway	6	1.2%
Cytoskeletal regulation by Rho GTPase	5	1.0%
Inflammation mediated by chemokine and cytokine	5	1.0%
Angiogenesis	4	0.8%
Huntington disease	3	0.6%
FAS signalling pathway	3	0.6%
Parkinson disease	3	0.6%
p53 pathway	3	0.6%
Apoptosis signalling pathway	3	0.6%
Nicotinic acetylcholine receptor signalling pathway	3	0.6%
CCKR signalling map	3	0.6%
Pentose phosphate pathway	3	0.6%
Muscarinic acetylcholine receptor 2 and 4 signalling pathway	2	0.4%
EGF receptor signalling pathway	2	0.4%
FGF signalling pathway	2	0.4%
Pyruvate metabolism	2	0.4%
B cell activation	2	0.4%
Dopamine receptor mediated signalling pathway	2	0.4%
Axon guidance mediated by semaphorins	2	0.4%
T cell activation	2	0.4%
Vitamin D metabolism and pathway	2	0.4%
Insulin/IGF pathway-protein kinase B signalling pathway	2	0.4%
Insulin/IGF pathway-mitogen activated protein kinase kinase/MAP kinase cascade	2	0.4%
Fructose galactose metabolism	1	0.2%
Pyrimidine Metabolism	1	0.2%
De novo purine biosynthesis	1	0.2%
Alzheimer disease-amyloid secretase pathway	1	0.2%
Angiotensin II-stimulated signalling through G proteins and beta-arrestin	1	0.2%
Muscarinic acetylcholine receptor 1 and 3 signalling pathway	1	0.2%
Adrenaline and noradrenaline biosynthesis	1	0.2%
Hypoxia response via HIF activation	1	0.2%
TGF-beta signalling pathway	1	0.2%
Toll receptor signalling pathway	1	0.2%
Nicotine pharmacodynamics pathway	1	0.2%
Cholesterol biosynthesis	1	0.2%
Oxidative stress response	1	0.2%
Heterotrimeric G-protein signalling pathway-rod outer segment phototransduction	1	0.2%
Androgen/estrogene/progesterone biosynthesis	1	0.2%
Heterotrimeric G-protein signalling pathway-Gi alpha and Gs alpha mediated pathway	1	0.2%
2-arachidonoylglycerol biosynthesis	1	0.2%
TCA cycle	1	0.2%
ATP synthesis	1	0.2%
Ubiquitin proteasome pathway	1	0.2%

## References

[1] Tietze C. (1957). Reproductive span and rate of reproduction among Hutterite women. Obstet. Gynecol. Surv..

[2] ESHRE Special Interest Group of Embryology and Alpha Scientists in Reproductive Medicine (2017). The Vienna consensus: Report of an expert meeting on the development of art laboratory performance indicators. Hum. Reprod. Open.

[3] Lewandowska A.E., Fel A., Thiel M., Czaplewska P., Łukaszuk K., Wiśniewski J.R., Ołdziej S. (2021). Compatibility of distinct label-free proteomic workflows in absolute quantification of proteins linked to the oocyte quality in human follicular fluid. Int. J. Mol. Sci..

[4] Zamah A.M., Hassis M.E., Albertolle M.E., Williams K.E. (2015). Proteomic analysis of human follicular fluid from fertile women. Clin. Proteom..

[5] Oh J.W., Kim S.K., Cho K.C., Kim M.S., Suh C.S., Lee J.R., Kim K.P. (2017). Proteomic analysis of human follicular fluid in poor ovarian responders during in vitro fertilization. Proteomics.

[6] Thomas P.D., Ebert D., Muruganujan A., Mushayahama T., Albou L.P., Mi H. (2022). PANTHER: Making genome-scale phylogenetics accessible to all. Protein Sci..

[7] Balaban B., Brison D., Calderon G., Catt J., Conaghan J., Cowan L., Ebner T., Gardner D., Hardarson T., Lundin K. (2011). The Istanbul consensus workshop on embryo assessment: Proceedings of an expert meeting. Hum. Reprod..

[8] Szklarczyk D., Gable A.L., Lyon D., Junge A., Wyder S., Huerta-Cepas J., Simonovic M., Doncheva N.T., Morris J.H., Bork P. (2019). STRING v11: Protein–protein association networks with increased coverage, supporting functional discovery in genome-wide experimental datasets. Nucleic Acids Res..

[9] Vieira M., Saraiva M.J. (2014). Transthyretin: A multifaceted protein. Biomol. Concepts.

[10] Bernal J. (2017). Thyroid hormone regulated genes in cerebral cortex development. J. Endocrinol..

[11] Balmer J.E., Blomhoff R. (2002). Gene expression regulation by retinoic acid. J. Lipid Res..

[12] Moolenaar W.H., Houben A.J., Lee S.J., van Meeteren L.A. (2013). Autotaxin in embryonic development. Biochim. Et Biophys. Acta (BBA)—Mol. Cell Biol. Lipids.

[13] Hartl F.U., Bracher A., Hayer-Hartl M. (2011). Molecular chaperones in protein folding and proteostasis. Nature.

[14] Pla I., Sanchez A., Pors S.E., Pawlowski K., Appelqvist R., Sahlin K.B., Poulsen L.L.C., Marko-Varga G., Andersen C.Y., Malm J. (2021). Proteome of fluid from human ovarian small antral follicles reveals insights in folliculogenesis and oocyte maturation. Hum. Reprod..

[15] Zhang X., Xu X., Li P., Zhou F., Kong L., Qiu J., Yuan Z., Tan J. (2019). TMT based proteomic analysis of human follicular fluid from overweight/obese and normal-weight patients with polycystic ovary syndrome. Front. Endocrinol..

[16] Bianchi L., Gagliardi A., Landi C., Focarelli R., De Leo V., Luddi A., Bini L., Piomboni P. (2016). Protein pathways working in human follicular fluid: The future for tailored IVF?. Expert Rev. Mol. Med..

[17] Karimpour-Fard A., Epperson E., Hunter L. (2015). A survey of computational tools for downstream analysis of proteomic and other omic datasets. Hum. Genom..

[18] Lee J.W., Lee J., Park M., Song S. (2005). An extensive comparison of recent classification tools applied to microarray data. Comput. Stat. Data Anal..

[19] Wu B., Abbott T., Fishman D., McMurray W., Mor G., Stone K., Ward D., Williams K., Zhao H. (2003). Comparison of statistical methods for classifcation of ovarian cancer using mass spectrometry data. Bioinformatics.

[20] Geurts P., Fillet M., Seny D., Meuwis M.A., Malaise M., Merville M., Wehenkel L. (2005). Proteomic mass spectra classification using decision tree based ensemble methods. Bioinformatics.

[21] Amaratunga D., Cabrera J., Lee Y.S. (2008). Enriched random forests. Bioinformatics.

[22] Saeys Y., Inza I., Larranaga P. (2007). A review of feature selection techniques in bioinformatics. Bioinformatics.

[23] Altmann A., Tolosi L., Sander O., Lengauer T. (2010). Permutation importance: A corrected feature importance measure. Bioinformatics.

[24] Strobl C., Boulesteix A.L., Kneib T., Augustin T., Zeileis A. (2008). Conditional Variable Importance for Random Forests. BMC Bioinform..

[25] World Health Organization (2010). WHO Laboratory Manual for the Examination and Processing of Human Semen.

[26] Lukaszuk K., Kunicki M., Liss J., Lukaszuk M., Jakiel G. (2013). Use of ovarian reserve parameters for predicting live births in women undergoing in vitro fertilization. Eur. J. Obstet. Gynecol. Reprod. Biol..

[27] Barritt J., Kokot M., Cohen J., Steuerwald N., Brenner C. (2002). Quantification of human ooplasmic mitochondria. Reprod. Biomed. Online.

[28] Wiśniewski J.R. (2016). Quantitative evaluation of filter aided sample preparation (FASP) and multienzyme digestion FASP protocols. Anal. Chem..

[29] Rappsilber J., Mann M., Ishihama Y. (2007). Protocol for micro-purification, enrichment, pre-fractionation and storage of peptides for proteomics using StageTips. Nat. Protoc..

[30] Gillet L.C., Navarro P., Tate S., Röst H., Selevsek N., Reiter L., Bonner R., Aebersold R. (2012). Targeted data extraction of the MS/MS spectra generated by data-independent acquisition: A new concept for consistent and accurate proteome analysis. Mol. Cell. Proteom..

[31] Zhang Y., Bilbao A., Bruderer T., Luban J., Strambio-De-Castillia C., Lisacek F., Hopfgartner G., Varesio E. (2015). The use of variable Q1 isolation windows improves selectivity in LC–SWATH–MS acquisition. J. Proteome Res..

[32] Breiman L. (2001). Random forests. Mach. Learn..

[33] Guyon I., Weston J., Barnhill S., Vapnik V. (2002). Gene Selection for Cancer Classification Using Support Vector Machines. Mach. Learn..

[34] Darst B., Malecki K., Engelman C. (2018). Using recursive feature elimination in random forest to account for correlated variables in high dimensional data. BMC Genet..

[35] Jeon H., Oh S. (2020). Hybrid-Recursive Feature Elimination for Efficient Feature Selection. Appl. Sci..

[36] Chen Q., Meng Z., Liu X., Jin Q., Su R. (2018). Decision Variants for the Automatic Determination of Optimal Feature Subset in RF-RFE. Genes.

